# Behavioral Responses and Contact Toxicity of Australian Tea Tree Oil and Its Major Constituents Against the Asian Citrus Psyllid, *Diaphorina citri* Kuwayama

**DOI:** 10.3390/insects17040355

**Published:** 2026-03-24

**Authors:** Fengmei Yang, Yuyun Liao, Yanjun Guo, Ranran Nie, Yourong Fu, Bingkun Chen, Qiwei Zhang, Qianhua Ji

**Affiliations:** 1 Fruit Tree Research Institute, Zhaoqing University, Zhaoqing 526061, China; fm_yang0801@163.com; 2 School of Life Sciences, Zhaoqing University, Zhaoqing 526061, China; 13680805491@163.com (Y.L.);

**Keywords:** *Melaleuca alternifolia*, essential oil, *Diaphorina citri*, contact toxicity, repellency, Terpinen-4-ol

## Abstract

The Asian citrus psyllid *Diaphorina citri* Kuwayama (Hemiptera: Liviidae) is the vector of the devastating citrus disease Huanglongbing, posing a significant threat to the global citrus industry and necessitating environmentally sound management strategies. This study aimed to evaluate Australian tea tree oil (TTO) and its primary constituents as potential botanical insecticides. Gas chromatography-mass spectrometry (GC-MS) was performed to analyze the chemical profile of commercial TTO, and behavioral effects on *D. citri* adults were assessed using a Y-tube olfactometer. Direct spray bioassays were conducted to determine contact toxicity. A total of 12 compounds were identified, with TTO being a Terpinen-4-ol chemotype, dominated by Terpinen-4-ol (40.62%), γ-Terpinene (21.46%), and α-Terpinene (10.45%). TTO demonstrated potent, concentration-dependent repellency, achieving 100% repellency at 10 g/L. In contrast, Terpinen-4-ol alone was attractive to psyllids at low concentrations, suggesting synergistic or masking effects within the complex oil blend. TTO and its major constituents also exhibited significant dose- and time-dependent contact toxicity. Although the 72 h LC_50_ of TTO (19.18 g/L) indicates lower potency compared to conventional insecticides (0.59–1.23 g/L), its combined repellent and toxic properties make it a promising candidate for integrated pest management (IPM) programs aimed at controlling *D. citri* and mitigating insecticide resistance.

## Simple Summary

The Asian citrus psyllid, *Diaphorina citri*, is a major threat to citrus farming worldwide because it spreads the devastating plant disease Huanglongbing (citrus greening). To protect citrus trees, farmers rely heavily on chemical pesticides, which can harm the environment and become less effective as insects develop resistance. This study explored a more natural solution: Australian tea tree oil (TTO). We aimed to understand the oil’s chemical makeup and test its ability to repel and kill this harmful psyllid. Our results showed that the complete oil is a powerful repellent. Interestingly, its most abundant component, Terpinen-4-ol, attracted the psyllids at low concentrations, suggesting the oil’s effectiveness comes from a complex blend of its ingredients. The oil also proved toxic to the psyllids on contact. Although not as fast-acting as synthetic chemicals, our findings show that TTO is a promising, environmentally friendly alternative that could be integrated into pest control programs to manage the Asian citrus psyllid.

## 1. Introduction

Huanglongbing (HLB), or citrus greening, is one of the most destructive diseases of citrus worldwide. To date, over 10 provinces in China have reported severe damage to citrus orchards by HLB, with annual losses estimated at 2 million tons of citrus fruits [1]. The disease is primarily vectored by the Asian citrus psyllid, *Diaphorina citri* Kuwayama (Hemiptera: Liviidae), a globally significant pest capable of spreading the infection year-round and inflicting enormous economic losses [2]. Currently, management of *D. citri* relies heavily on frequent applications of chemical insecticides, including neonicotinoids, pyrethroids, and avermectins [3]. However, this intensive use has led to significant environmental risks, including pesticide residues, harm to non-target organisms, and the widespread development of insecticide resistance in *D. citri* populations to major chemical classes, particularly neonicotinoids and pyrethroids, as documented in field studies [4,5,6]. Consequently, there is an urgent need to explore sustainable and ecologically sound alternatives, such as plant-derived essential oils, for integrated pest management (IPM).

Plant essential oils are complex mixtures of volatile secondary metabolites that are promising natural alternatives to synthetic pesticides. They can directly inhibit pests through toxic, antifeedant, or repellent mechanisms, or indirectly by attracting natural enemies [7]. Previous studies have validated the role of various essential oils in *D. citri* and other pest control strategies. For instance, guava essential oil alters the feeding behavior of *D. citri*, reducing host damage [8], while the floral essential oil of *Praxelis clematidea* functions as a repellent and fumigant against this pest [9]. A systematic evaluation of seven plant essential oils for their fumigant toxicity and repellency against the red flour beetle, *Tribolium castaneum*, identified Mexican tea and native Indian palm oils as highly effective repellents [10]. A previous study also revealed that essential oils from *Citrus reticulata* and *Melaleuca alternifolia* exhibited significant efficacy in inhibiting *Drosophila suzukii* oviposition and acting as repellents [11].

*Melaleuca alternifolia*, commonly known as tea tree, is a fast-growing plant in the Myrtaceae family with a high oil yield [12]. Essential oils from the Myrtaceae family, particularly from the genera *Eucalyptus* and *Melaleuca*, have demonstrated high insecticidal potential due to their rich terpene content [13]. The essential oil extracted from its leaves and shoots, known as tea tree oil (TTO), is a natural product with a pleasant aroma and well-documented antibacterial and anti-inflammatory [12]. Although TTO has been widely studied for medical and industrial applications, its potential for controlling *D. citri* remains largely unexplored. Our preliminary field observations indicated that the distinct aroma of TTO has a repellent effect on *D. citri*, prompting a more systematic investigation. This study therefore aimed to: (1) characterize the chemical composition of a commercial TTO, and (2) investigate the behavioral and toxicological effects of the oil and its major constituents on *D. citri* adults. This work is anticipated to provide a scientific basis for developing novel, green management strategies for *D. citri* using TTO.

## 2. Materials and Methods

### 2.1. Materials

#### 2.1.1. Plants and Reagents

*Melaleuca alternifolia* and *Murraya exotica* plants were cultivated in a screen house at the Fruit Tree Research Institute of Zhaoqing University, China, with sufficient light and water, a soil pH maintained between 4.5 and 6.5, and an average annual temperature of 19–22 °C. The pure TTO (99.99%) was extracted from the leaves of these 4–5-year-old *M. alternifolia* trees of the Terpinen-4-ol chemotype, and purchased from Jiangxi Xuesong Natural Medicinal Oil Co., Ltd. (Ji’an, China). Analytical standards of Terpinen-4-ol (98% purity), γ-Terpinene (98%), α-Terpinene (98%), α-Terpineol (98%), and p-Cymene (98%) were purchased from Beijing Jingdong Industrial Products Trading Co., Ltd. (Beijing, China). Tween-80 and analytical grade absolute ethanol were also acquired from this supplier. Commercial emulsifiable concentrate formulations of 5% Abamectin, 5% λ-Cyhalothrin, and a 20% Biphenyl·thiamethoxam mixture were purchased from Zhengzhou Jinyunong Agricultural Technology Co., Ltd. (Zhengzhou, China).

#### 2.1.2. Insect Rearing

The *Diaphorina citri* colony was originally collected from fingered citron (*Citrus medica* var. *sarcodactylis*) plants in an abandoned orchard in Dinghu District, Zhaoqing City. The population was subsequently maintained on fresh *M. exotica* shoots in a screen house at the Fruit Tree Research Institute of Zhaoqing University, and had no prior exposure to any chemical insecticides. All bioassays were conducted using mixed-sex adult psyllids, 3–5 days after eclosion. Insects were starved for 2 h before testing.

### 2.2. Methods

#### 2.2.1. Gas Chromatography-Mass Spectrometry (GC-MS) Analysis

The TTO sample was sent to Shanghai Weipu Inspection Technology Group Co., Ltd. (Shanghai, China) for compositional analysis by GC-MS. The analysis was performed on an Agilent GC-MS system (Agilent Technologies, Santa Clara, CA, USA) (GC: 7890B, MS: 5977A) equipped with a TG-5 ms capillary column (30 m × 0.25 mm × 0.25 μm). Helium was used as the carrier gas at a flow rate of 1 mL/min. The injector temperature was 220 °C, and 1 μL of the sample was injected with a split ratio of 50:1. The oven temperature was programmed as follows: initial temperature of 60 °C held for 2 min, ramped to 120 °C at 4 °C/min, then to 180 °C at 2 °C/min, and finally to 230 °C at 10 °C/min, with no hold time at the end of each ramp. The solvent delay was 3 min. The MS was operated in electron ionization (EI) mode with an ion source temperature of 230 °C. The mass scan range was *m*/*z* 30–450.

Compounds were identified by comparing their mass spectra with those in the NIST 2017 library. Major terpenoids were identified by comparing their retention times and mass spectra with those of authentic standards (in n-hexane) under the same conditions, with three biological replicates performed for each sample. The relative content of each component was quantified by the peak area normalization method.

#### 2.2.2. Preparation of Test Solutions

Preliminary experiments were conducted to evaluate the emulsifying efficacy of Tween-80 at concentrations of 0.1%, 0.2%, 0.3%, 0.4%, 0.5%, and 0.6% (*v*/*v*) with TTO. The 0.5% (*v*/*v*) Tween-80 solution was found to form a stable and uniform oil-in-water emulsion and is widely used in bioassays of botanical insecticides to effectively emulsify hydrophobic essential oils without exhibiting toxicity to target insects [14]. Accordingly, a 0.5% (*v*/*v*) aqueous solution of Tween-80 was prepared using sterile distilled water to serve as the surfactant and control. Stock solutions of TTO and its five major individual components were prepared by emulsifying a specific volume of the oil or compound in the 0.5% Tween-80 solution, followed by vigorous vortexing for 1 min to create stable emulsions of the desired concentrations. All test emulsions were freshly prepared for immediate use to ensure consistency in concentration and state for each behavioral test.

#### 2.2.3. Behavioral Assay

**Figure 1:**
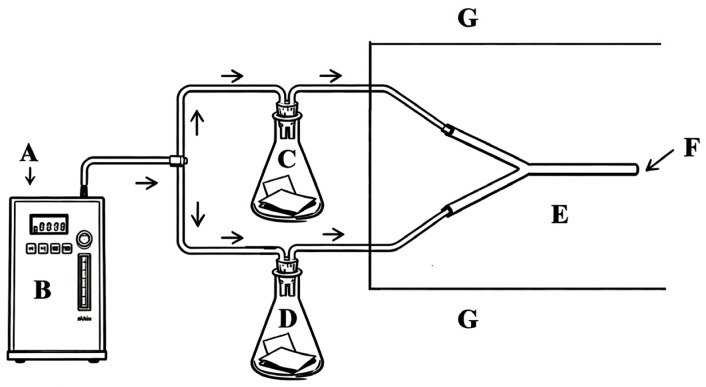
The diagram of the olfactometer. A, Air source; B, Atmospheric sampler; C, Erlenmeyer flask containing treatment odors; D, Erlenmeyer flask containing control odors; E, Y-tube olfactometer; F, Release point of adult psyllids; G, Dark treatment with black cloth.

The behavioral responses of adult *D. citri* were assessed using a glass Y-tube olfactometer (main arm: 15 cm; side arms: 15 cm at a 70° angle; internal diameter: 1.5 cm), custom-made by Henan Scientific Instrument Factory (Zhengzhou, China). The setup consisted of an air source, an atmospheric sampler (QC-1B, Beijing Ke’an Labor Protection New Technology Co., Beijing, China), two sample bottles (for treatment and control), and the Y-tube (Figure 1). An opaque black cloth was placed over the olfactometer to eliminate interference from external light sources.

Purified and humidified air was drawn through the two sample bottles and into the corresponding arms of the olfactometer at a total flow rate of 20 mL/min. For each trial, a filter paper treated with the diluted TTO emulsion was placed in one sample bottle (treatment arm), while a filter paper treated with 0.5% Tween-80 solution was placed in the other bottle (control arm). The system was aerated for 30 min before introducing the insects.

For each replicate, 15 vigorous adult psyllids were introduced individually into the main arm. A choice was recorded when a psyllid moved more than 2.5 cm into either arm and remained there for at least 1 min, timed with a stopwatch. Psyllids that did not make a choice within 5 min were recorded as non-responders. Each concentration was tested with three independent replicates (total *n* = 45). To avoid positional bias, the positions of the treatment and control arms were swapped after each replicate. The glassware was cleaned with 75% ethanol and rinsed with distilled water between replicates, followed by air-drying. The repellency rate was calculated as follows: Repellency Rate (%) = (Number of insects in control arm/Total number of responding insects) × 100%.

#### 2.2.4. Contact Toxicity Assay

Based on preliminary range-finding experiments, TTO was diluted to 20, 40, 60, 80, and 100 g/L. The five major components and three conventional insecticides were diluted as shown in Table 1. For each replicate, 15 active adult psyllids were collected from the screen house using a bottle (a modified 150 mL transparent plastic bottle, 52 mm diameter × 72 mm height, with a 34 mm outer opening diameter) and kept inside. The bottle was then sealed with a 200-mesh screen. A handheld fine-mist sprayer was used to apply 2 mL of the test solution into the bottle from a fixed distance of 5 cm. After the solution on the insects had air-dried (approx. 10 min), a fresh, untreated branch of *M. exotica* was placed in the bottle as a food source. The control group was treated with a 0.5% Tween-80 solution. Each treatment was replicated three times. The bottles were maintained in an artificial climate chamber at 25 ± 1 °C and 65 ± 5% RH. Mortality was recorded at 24, 48, and 72 h post-treatment. Psyllids were considered dead if they did not move when prodded with a fine brush. The experiment was considered valid if mortality in the control group was ≤20%. Raw mortality was calculated as Mortality (%) = (Number of dead insects/Total number of insects) × 100%. Psyllid mortality was then corrected using Abbott’s formula: Corrected Mortality (%) = [(Treatment mortality − Control mortality)/(100 − Control mortality)] × 100%.

#### 2.2.5. Data Processing and Analysis

Data were organized and processed using Microsoft Excel 2019. In the Y-tube olfactometer bioassay, the number of psyllids choosing the treatment arm versus the control arm was analyzed using a chi-square (χ^2^) goodness-of-fit test and Fisher’s exact test to determine if the observed distribution deviated significantly from an expected 50:50 ratio. For the contact toxicity assays, mortality data were subjected to probit analysis using SPSS Statistics 26 (IBM Corp., Armonk, NY, USA) to calculate the 50% lethal concentration (LC_50_), 95% confidence intervals, and the slope of the dose–response line. Toxicity data and behavioral responses were visualized using GraphPad Prism 9.5.0 (GraphPad Software, San Diego, CA, USA).

## 3. Results

### 3.1. Chemical Composition of TTO

GC-MS analysis of the commercial TTO identified 12 compounds with relative abundances > 0.2%, accounting for 99.99% of the total oil composition (Table 2). The oil was primarily composed of oxygenated monoterpenes (49.96%) and monoterpene hydrocarbons (47.85%). The most abundant compound was Terpinen-4-ol (40.62%), followed by γ-Terpinene (21.46%) and α-Terpinene (10.45%). The five most abundant compounds—Terpinen-4-ol, γ-Terpinene, α-Terpinene, p-Cymene (6.33%), and α-Terpineol (6.40%)—accounted for over 85% of the total oil. Based on the high concentration of Terpinen-4-ol (>30%) and low concentration of 1,8-Cineole (<5%), the TTO was confirmed to be of the Terpinen-4-ol chemotype, consistent with international standards (ISO 4730:2017) [15] and the national standard of China (GB/T 26514-2011) [16].

### 3.2. Behavioral Effects of TTO and Its Major Components on Adult D. citri

**Figure 2:**
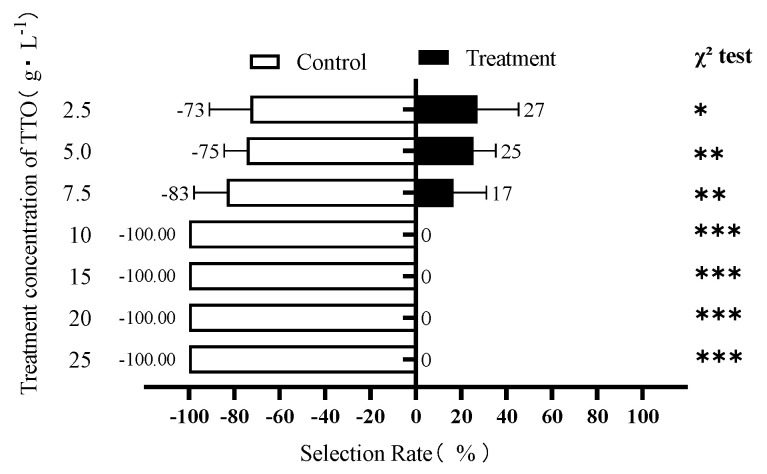
Behavioral response of *D. citri* adults to different concentrations of TTO in a Y-tube olfactometer. Bars represent the percentage of psyllids choosing the treatment (TTO) or control (CK) arm. Error bars represent the Standard Error of the Mean (SEM) of three replicates. Asterisks indicate a significant difference between the number of insects choosing the treatment versus control arm within a concentration (Chi-square test: * *p* < 0.05; ** *p* < 0.01; *** *p* < 0.001).

In the Y-tube olfactometer assay, TTO exhibited a significant, concentration-dependent repellent effect on adult *D. citri* (Figure 2). Repellency increased with concentration, reaching 100% at concentrations of 10 g/L and above, where all psyllids chose the control arm (mean = 13 ± 1; χ^2^ = 26.00, df = 1, *p* < 0.0001).

**Figure 3:**
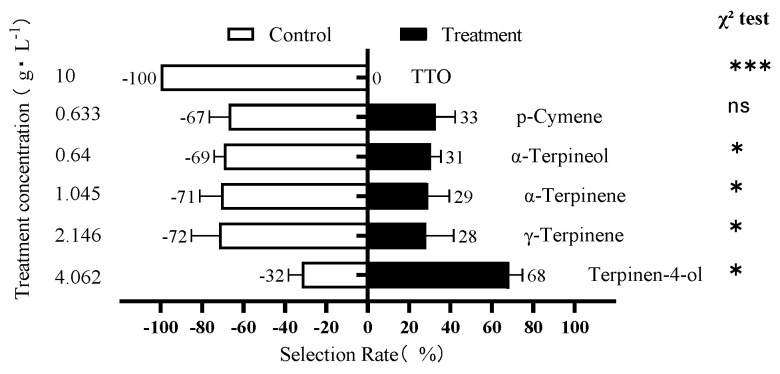
Behavioral response of *D. citri* adults to 10 g/L TTO and its five major components at their corresponding concentrations. Bars represent the percentage of psyllids choosing the treatment or control (CK) arm. Error bars represent SEM of three replicates. Asterisks indicate a significant difference (Chi-square test: * *p* < 0.05; *** *p* < 0.001; ns, not significant).

Based on these results, the behavioral effects of TTO at 10 g/L and its five major components at their corresponding concentrations were compared (Figure 3). TTO at 10 g/L was strongly repellent (100%). γ-Terpinene and α-Terpinene were also significantly repellent, with repellency rates of 72% (mean = 11 ± 1; χ^2^ = 3.86, df = 1, *p* < 0.05) and 71% (mean = 13 ± 1; χ^2^ = 5.76, df = 1, *p* < 0.02). In stark contrast, Terpinen-4-ol at its corresponding concentration (4.06 g/L) was significantly attractive, with 68% of psyllids choosing the treatment arm (mean = 13 ± 2; χ^2^ = 4.24, df = 1, *p* < 0.05). α-Terpineol and p-Cymene did not elicit a significant attractive or repellent response at the tested concentrations.

### 3.3. Contact Toxicity of TTO and Its Components Against D. citri

**Figure 4:**
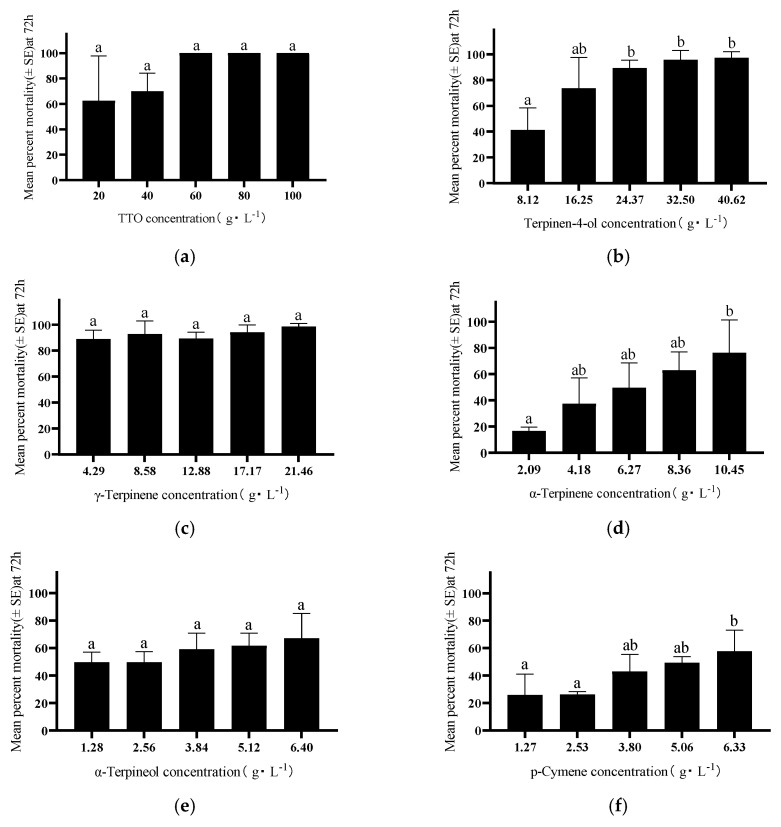
Contact toxicity of TTO and its five major components against *D. citri* adults within 72 h: (**a**) TTO; (**b**) Terpinen-4-ol; (**c**) γ-Terpinene; (**d**) α-Terpinene; (**e**) α-Terpineol; (**f**) p-Cymene. Different lowercase letters above the bars indicate significant differences among treatments (*p* < 0.05, Tukey’s HSD test).

TTO and its five major components exhibited concentration- and time-dependent contact toxicity against *D. citri* adults (Figure 4). The toxicity of TTO and its components was significantly higher than the control at all time points (24, 48, and 72 h). Among the compounds, TTO was the most toxic, followed by Terpinen-4-ol and γ-Terpinene. After 72 h, mortality rates exceeded 70% for TTO at ≥40 g/L, Terpinen-4-ol at ≥16.25 g/L, γ-Terpinene at ≥4.29 g/L, and α-Terpinene at ≥10.45 g/L, demonstrating their potent toxic effects.

### 3.4. Contact Toxicity of TTO vs. Conventional Insecticides

**Figure 5:**
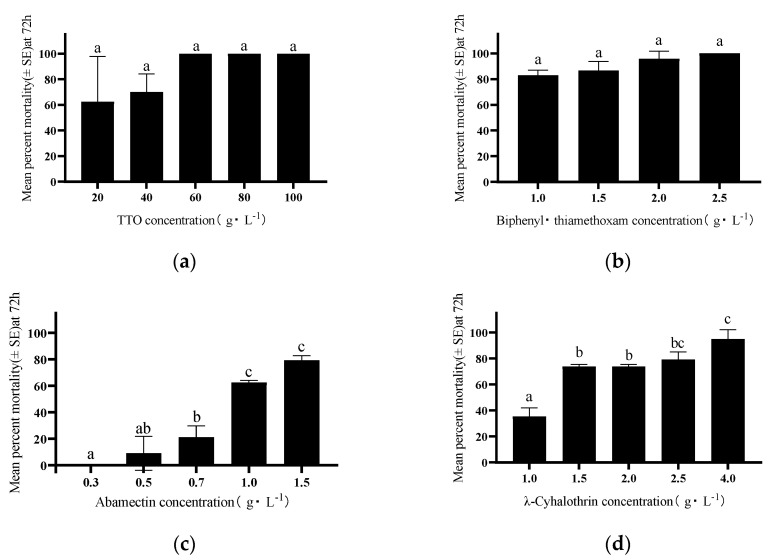
Contact toxicity of TTO and three common chemical insecticides against *D. citri* adults within 72 h: (**a**) TTO; (**b**) Biphenyl·thiamethoxam; (**c**) Abamectin; (**d**) λ-Cyhalothrin. Different lowercase letters above the bars indicate significant differences among treatments (*p* < 0.05, Tukey’s HSD test).

TTO and the three conventional insecticides all exhibited significant dose- and time-dependent contact lethality against *D. citri* adults (Figure 5). The Biphenyl·thiamethoxam mixture was highly lethal (81.28% mortality) at a low concentration (2.5 g/L) within 24 h of exposure. Similarly, TTO demonstrated good efficacy (>85.5% mortality) at higher concentrations (≥80 g/L) after 24 h of exposure. After 72 h, the mortality rates for 40 g/L TTO, 1.0 g/L Biphenyl·thiamethoxam, 1.5 g/L Abamectin, and 1.5 g/L λ-Cyhalothrin were 70.00%, 82.86%, 79.37% and 73.86%, respectively, indicating effective contact control at these concentrations.

As shown in Table 3, the toxicity of all tested agents increased over time. Based on the 72 h LC_50_ values, the order of toxicity was: Biphenyl·thiamethoxam (0.59 g/L) > Abamectin (0.93 g/L) > λ-Cyhalothrin (1.23 g/L) > TTO (19.18 g/L). Biphenyl·thiamethoxam was the most potent insecticide, being approximately 32.5 times more toxic than TTO after 72 h.

## 4. Discussion

### 4.1. Variation in Essential Oil Composition and Activity

The chemical composition of *M. alternifolia* essential oil can vary based on geography, harvest time, and extraction method, leading to different chemotypes [17,18]. Among the constituents of TTO identified here, Terpinen-4-ol (40.62%) was paramount and 1,8-cineole (2.92%) remained minimal, aligning with standard Terpinen-4-ol chemotypes. Terpinen-4-ol is the dominant compound in *M. alternifolia*, which is consistent with many previous reports [17,19]. Terpinen-4-ol has consistently demonstrated strong biological activities, including insecticidal effects against the red flour beetle and maize weevil through enzyme inhibition and fumigant toxicity [20]. The combined presence of Terpinen-4-ol and highly volatile hydrocarbons (γ-Terpinene, α-Terpinene) in TTO likely generates the potent synergistic toxicity observed in our bioassays. The superior overall efficacy of the complete oil compared to its individual components strongly points towards a synergistic action among the constituents, a phenomenon also reported for other essential oils against different pests [21].

### 4.2. Differential Repellent and Toxic Activities

Our study indicates that TTO exerts significant repellent and lethal activities against *D. citri* in a dose- and time-dependent manner. TTO exhibited the strongest repellency (100%) against *D. citri* adults at concentrations ≥ 10 g/L. This corresponds closely with prior screening efforts. For example, Mann et al. (2012) tested various plant-based essential oils, observing that full-strength botanical blends induced superior repellent and avoidance behaviors in *D. citri* compared to single-component isolates [22]. Similarly, Ye et al. (2024) reported that Terpinen-4-ol-type *M. alternifolia* branches and leaves effectively repelled *D. citri* in field trials [17].

Notably, in the present study, TTO at ≥10 g/L and the individual components γ-Terpinene (2.15 g/L) and α-Terpinene (1.05 g/L) were highly repellant (>70% repellency). However, in contrast, Terpinen-4-ol, the most abundant component of TTO, was attractive to *D. citri* at 4.06 g/L. This discrepancy suggests that the behavioral response of *D. citri* to Terpinen-4-ol may be dose-dependent, acting as an attractant at lower concentrations while potentially becoming repellent at higher vapor saturation. The contrasting behavioral effects of TTO components can be plausibly explained by their physicochemical properties and the concept of blend synergy. The highly volatile monoterpene hydrocarbons (γ-Terpinene and α-Terpinene, both have a boiling point below 200 °C) likely act as primary repellents by rapidly creating an “odor barrier”. In contrast, the less volatile oxygenated monoterpene (Terpinen-4-ol, a boiling point of 212 °C) may only be perceived at low concentrations, potentially mimicking a host cue or a pheromone component, leading to attraction. The strong repellency of the complete TTO, despite the attractiveness of its main component, suggests a synergistic or masking effect where other constituents either enhance the repellency of the terpenes or mask the attractive nature of Terpinen-4-ol. Although, not explicitly emphasized in the literature, data from Ye et al. (2024) show that low concentrations of γ-Terpinene (0.1 μL/mL) in TTO can also exert a significant attractant effect [17]. However, these explanations remain hypothetical without further physiological (e.g., EAG/SSR) and detailed dose–response behavioral assays.

When comparing our toxicity results to previous work, Mann et al. (2012) reported LC_50_ values for *D. citri* adults ranging from 0.16 μg/insect (lavender oil) to 17.26 μg/insect (thyme oil) [22]. Although direct comparisons are constrained by differences in reporting units and bioassay methodology (our TTO LC_50_ was 19.18 g/L on adults), these findings confirm that plant-derived essential oils possess significant contact toxicity against this vector. Given the increasing development of resistance to conventional synthetic insecticides [4,5,6], our findings support the further exploration of TTO as a viable, albeit distinct, tool for integrated pest management programs.

### 4.3. Development Prospects of TTO

Our study confirms that TTO has significant repellent and lethal activities against *D. citri*, and its individual components exhibit attraction at lower concentrations, making TTO a promising candidate for development as a botanical insecticide or its components as attractants. Compared to conventional pesticides, essential oils like TTO offer the advantages of lower mammalian toxicity, higher biodegradability, and novel modes of action, which can be valuable in managing insecticide resistance [23].

However, recognizing the limitations of essential oils in field conditions is crucial. The contact assay in our study (utilizing 2 mL spray volumes) provides uniform target coverage that may not perfectly reflect variable field spraying efficiencies. Additionally, the high volatility and susceptibility to photodegradation of essential oil components often lead to poor persistence in the field [24,25]. Furthermore, the high LC_50_ value observed in our study indicates that relatively large amounts of TTO would be required for effective control, which may be cost-prohibitive. To overcome these limitations, future research should focus on developing advanced formulations, such as microencapsulations or nanoemulsions, to improve stability, enhance residual activity, and reduce the required dosage [26,27]. Integrating TTO-based products into a broader IPM strategy, possibly in rotation with other biopesticides or conventional insecticides, could provide a sustainable solution for *D. citri* management.

## Figures and Tables

**Table 1:** Concentrations of the test agents.

Treatment	Conc. 1 (g/L)	Conc. 2 (g/L)	Conc. 3 (g/L)	Conc. 4 (g/L)	Conc. 5 (g/L)
TTO	20	40	60	80	100
Terpinen-4-ol	8.12	16.25	24.37	32.5	40.62
γ-Terpinene	4.29	8.58	12.88	17.17	21.46
α-Terpinene	2.09	4.18	6.27	8.36	10.45
α-Terpineol	1.28	2.56	5.12	5.12	6.4
p-Cymene	1.27	2.53	3.8	5.06	6.33
λ-Cyhalothrin	1.0	1.5	2.0	2.5	4.0
Abamectin	0.3	0.5	0.7	1.0	1.5
Biphenyl·thiamethoxam	1.0	1.5	2.0	2.5	-

**Table 2:** Chemical composition of Australian tea tree oil (TTO).

No.	Retention Time (min)	Compound	Molecular Formula	Content (%)
1	6.174	Pinene	C_10_H_16_	3.81
2	8.579	α-Terpinene	C_10_H_16_	10.45
3	8.808	p-Cymene	C_10_H_14_	6.33
4	8.968	D-Limonene	C_10_H_16_	2.35
5	9.071	1,8-Cineole	C_10_H_18_O	2.92
6	9.918	γ-Terpinene	C_10_H_16_	21.46
7	10.814	Terpinolene	C_10_H_16_	2.75
8	14.105	Terpinen-4-ol	C_10_H_18_O	40.62
9	14.558	α-Terpineol	C_10_H_18_O	6.40
10	23.579	Aromadendrene	C_15_H_24_	2.18
11	25.902	Ledene	C_15_H_24_	0.49
12	30.215	Globulol	C_15_H_26_O	0.24

**Table 3:** Contact toxicity of TTO and three common insecticides against *D. citri* adults.

Treatment	Processing Time (h)	LC_50_ (g/L)	95% Confidence Interval (g/L)	Regression Equation (Y=)	χ^2^	df	*p*-Value	Correlation Coefficient (r)	Relative Toxicity (72 h)
TTO	24 h	46.72	33.93~57.52	−8.46 + 5.07x	2.09	3	0.55	1.00	0.03
	48 h	33.42	20.33~43.73	−5.92 + 3.89x	0.81	3	0.85	1.00	0.02
	72 h	19.18	3.18~28.57	−4.33 + 3.38x	3.17	3	0.37	0.89	0.03
λ-Cyhalothrin	24 h	3.10	2.41~5.40	−1.71 + 3.48x	0.85	3	0.84	1.00	0.45
	48 h	2.10	1.64~2.76	−1.18 + 3.66x	1.06	3	0.79	1.00	0.35
	72 h	1.23	0.72~1.57	−0.33 + 3.59x	1.61	3	0.66	1.00	0.48
Abamectin	24 h	1.77	1.29~10.68	−0.98 + 3.94x	0.36	3	0.95	0.98	0.80
	48 h	1.26	0.98~2.17	−0.38 + 3.80x	0.66	3	0.88	1.00	0.59
	72 h	0.93	0.76~1.22	0.14 + 4.40x	1.34	3	0.72	1.00	0.63
Biphenyl·thiamethoxam	24 h	1.41	0.66~1.81	−0.54 + 3.66x	0.86	2	0.65	1.00	1.00
	48 h	0.74	-	0.29 + 2.13x	1.63	2	0.44	0.80	1.00
	72 h	0.59	-	0.78 + 3.36x	0.65	2	0.72	1.00	1.00

Note: Relative Toxicity (72 h) calculated as (LC_50_ of most toxic agent/LC_50_ of test agent).

## Data Availability

The original contributions presented in this study are included in the article. Further inquiries can be directed to the corresponding authors.

## Abbreviations

The following abbreviations are used in this manuscript:

GC-MS	Gas chromatography-mass spectrometry
HLB	Huanglongbing
TTO	Tea tree oil
